# IgA vasculitis after COVID-19: a case-based review

**DOI:** 10.1007/s00296-024-05606-4

**Published:** 2024-05-13

**Authors:** Dorota Suszek, Anna Grzywa-Celińska, Justyna Emeryk-Maksymiuk, Adam Krusiński, Katarzyna Redestowicz, Jan Siwiec

**Affiliations:** 1 Department of Rheumatology and Connective Tissue Diseases, Medical University, Lublin, Poland; 2 Department of Pneumonology, Oncology and Allergology, Medical University, Lublin, Poland; 3 Department of Internal Medicine in Nursing, Medical University, Lublin, Poland

**Keywords:** COVID-19, Henoch-Schönlein purpura, IgA vasculitis, Palpable purpura, Prognosis

## Abstract

IgA-associated vasculitis (IgAV) known as Henoch - Schönlein purpura (HSP) disease is an inflammatory disorder of small blood vessels. It’s the most common type of systemic vasculitis in children which can be associated with the inflammatory process following infections. IgA vasculitis is a rare and poorly understood systemic vasculitis in adults. Coronavirus disease 2019 (COVID-19) has been associated with HSP in both adults and children. A 58-year-old woman was diagnosed with HSP, fulfilling the clinical criteria: palpable purpura, arthritis, hematuria. The disclosure of the HSP disease was preceded by a infection of the respiratory tract. COVID-19 infection was confirmed via the presence of IgM and IgG antibodies. This case indicates the possible role of SARS-CoV-2 in the development of HSP. The clinical course of IgAV in adults appears to be different from pediatric IgAV, especially due to higher risk of renal complications. Symptoms of the disease quickly resolved with low-dose of steroids.

## Introduction

IgA vasculitis (IgAV), also known as Henoch-Schönlein purpura (HSP), is an inflammation of small vessels with deposition of immunoglobulin class A (IgA) in the blood vessels of the skin and internal organs. IgAV/HSP is the most common form of small vessel vasculitis in children, 10 times less common in adults. The annual incidence is about 15/100,000 in children and 1.3/100,000 in adults [1–3]. The etiology of IgAV/HSP is not fully understood. There is an association between IgAV and a history of bacterial infection, viral infection, vaccination, food allergens, and drug use. More than a dozen cases of IgAV after COVID-19 have recently been described [4]. A defect in the glycosylation of immunoglobulin IgA1 and its abnormal structure are basic phenomena in IgAV. Immunoglobulin IgA1 does not contain galactose molecules attached during glycosylation in its hinge region (galactose-deficient IgA1: Gd-IgA1). Abnormal IgA1 is formed after contact with antigens on the surface of mucous membranes of the upper respiratory tract and gastrointestinal tract [5]. The presence of IgA1 in blood induces the formation of autoantibodies, immune complexes. The pathogenesis of IgAV in the course of COVID-19 is associated with abnormal development of cellular response (Th2), increased activation of B cells and production of antibodies. This leads to type 3 hypersensitivity reactions, deposition of antigen-antibody complexes, usually in blood vessels, activation of the complement cascade and release of anaphylatoxins (C3a and C5a) [6]. Cytokines released during COVID-19 infection (IL-1, IL-6 and TNF) can also lead to B-cell proliferation and IgA1 production [7]. COVID-19 infection can lead to the development of IgAV after direct viral infection of endothelial cells via angiotensin-converting enzyme 2 receptors or indirectly via the inflammation induced by an immune response [8, 9]. The main symptoms of the disease are palpable purpura, arthritis, abdominal pain and glomerulonephritis. The diagnosis of IgAV is established on the basis of clinical symptoms and histopathological presentation of a skin or kidney specimen. According to the current SHARE (Single Hub and Access point for pediatric Rheumatology in Europe) guidelines, the 2010 EULAR/PRINTO/PRES (European Alliance of Associations for Rheumatology - EULAR, Pediatric Rheumatology International Trials Organization - PRINTO, Pediatric Rheumatology European Society - PRES) criteria are used to diagnose IgAV. These criteria are usually used for the diagnosis of IgAV in children and are of limited value in adult patients. However, they are more sensitive and specific than the 1990 ACR IgAV criteria. To diagnose IgAV, the presence of purpura and at least 1 of four symptoms: abdominal pain, arthritis/pain, renal involvement or histopathologically confirmed IgA-mediated vasculitis/glomerulonephritis is required [10, 11]. IgA vasculitis is a mild, self-limiting disease, especially in children. The course of the disease in adults is more severe, usually complicated by gastrointestinal and renal involvement. Gastrointestinal vasculitis can lead to intestinal perforation, hemorrhage and is the main risk of IgAV death in adults. Renal involvement is associated with an increased risk of progression to chronic renal failure [12, 13]. More than a dozen cases of IgAV after COVID-19 have been described so far. This virus can attack almost any organ and lead to skin lesions, renal, cardiovascular and neurological complications [14, 15].

Our study aims to conduct a review of case reports on IgAV caused by COVID-19. We will assess how the age of onset affects the clinical spectrum of the disease and compare the symptoms of IgAV in COVID-19 cases with those caused by other factors. Identification of this patient group may enable us to investigate prognostic factors and develop suitable therapeutic methods in the future. So far, the lack reviews on this topic.

## Case study

**Fig. 1:**
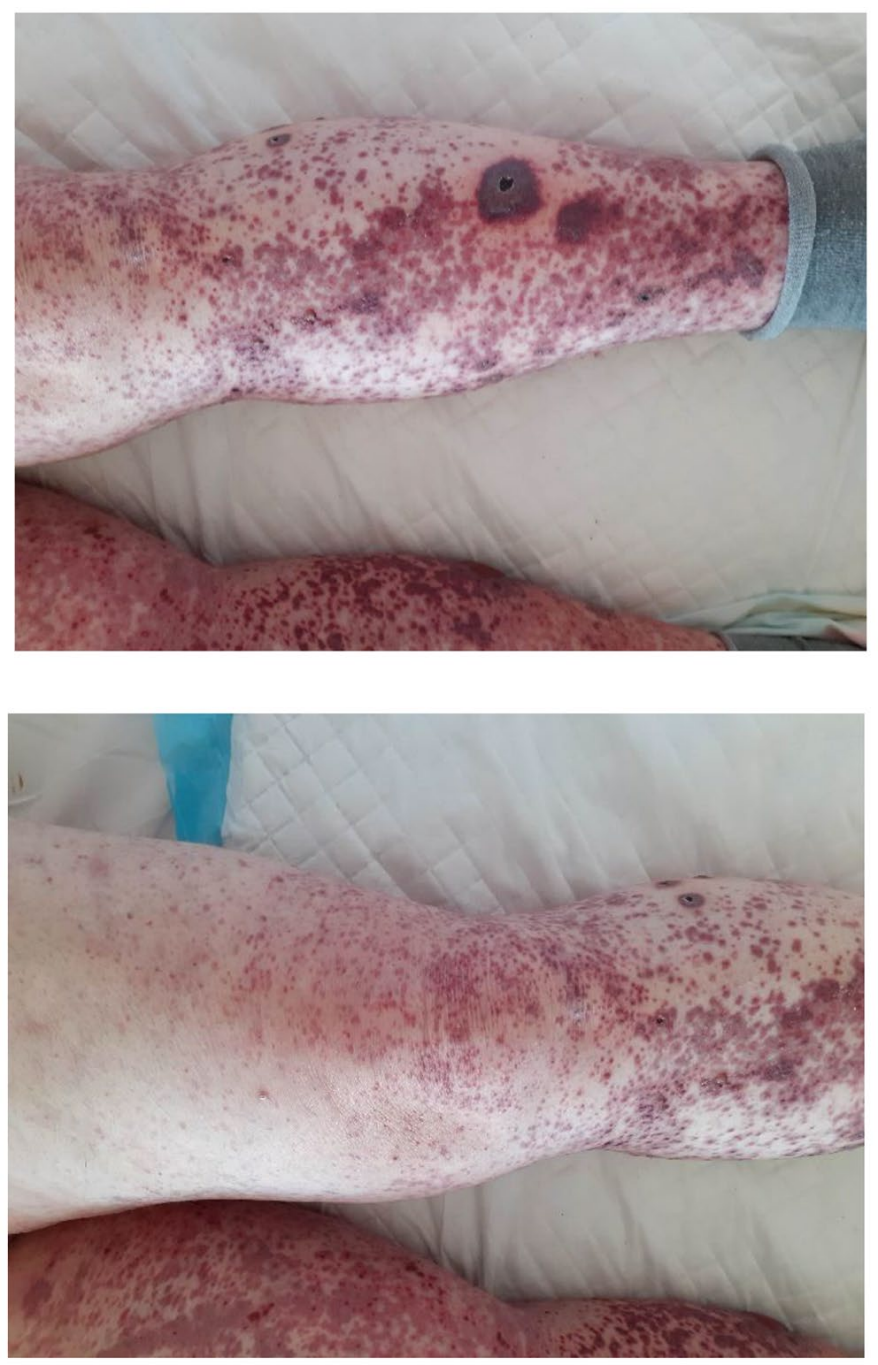
Palpable skin rash on the legs

The patient, 58 years old woman, was brought to the ED in December 2021 because of worrisome skin lesions (Fig. 1). A few days earlier, the patient had symptoms of respiratory tract infection: dry cough, subfebrile states. She was not taking antibiotics. Five days after the symptoms of infection, she developed a rash on the skin of the lower extremities and forearms with the character of raised purpura, pain and swelling of the left knee joint. The patient denied abdominal pain, hematuria, gastrointestinal bleeding. Test results showed: elevated inflammatory markers, thrombocytosis, leukocyturia and erythrocyturia (Table 1). Lung CT showed diffuse foci of lung parenchymal consolidation located subpleurally with bronchial lumen dilatation in segment 3 of both lungs, the right lower lobe and in segments 4P, 8 L, 9 L, as well as features of peripheral pulmonary embolism. An abdominal ultrasound showed no abnormalities. A positive antibody result for COVID-19 and a positive PCR test were obtained. The patient had not been vaccinated against COVID-19. A diagnosis of pneumonia due to COVID-19 infection was made, and the patient was transferred to the Isolation Unit. During hospitalization, virological and immunological diagnostics were performed (Table 2). On the basis of clinical symptoms and laboratory results: raised purpura, arthritis, hematuria, sterile leukocyturia, IgAV was diagnosed. The woman did not consent to a skin biopsy. The patient met the criteria for the diagnosis of the disease according to EULAR/PRINTO/PRES (2010). Treatment included glucocorticoids (GCs) methylprednisolone at the initial dose of 16 mg/once a day (after 3 days the dose was reduced to 8 mg/once a day), low molecular weight heparin at a dose of 1 mg/kg body weight/twice a day and then rivaroxaban at a dose of 15 mg/twice a day. The patient did not require oxygen therapy. After 10 days of treatment, a significant reduction in skin lesions was achieved. Further observation of the patient was carried out in the setting of a rheumatology outpatient clinic where the dose of GCs was reduced. After 2 months, complete remission of skin and joint symptoms, normalization of laboratory test results was achieved. GCs were discontinued.

**Table 1:** Laboratory data

Parameter	At the beginning of hospitalization	At the end of hospitalization
Hemoglobin (g/dl)	12.2	11.8
Total leukocyte count (cells/mcL)	6300	4200
Neutrophils (%/cells/mcL)	72.1/4500	52.7/2200
Lymphocytes (%/cells/mcL)	17.7/1130	35.3/1500
Platelets (cells/mcL)	537 000	330 000
Erythrocyte Sedimentation Rate (mm/1h) < 10	27	
C-Reactive Protein (mg/L) 0–5	10.5	0.3
Procalcitonin (ng/mL) < 0.5	< 0.02	0.08
IL- 6 (pg/ml) < 4.4	8.4	2.7
Erytrocyturia	20–25	1–3
Leukocyturia	15–20	1–3
24 h urinary protein (mg/24 h)	950	
Urine culture	negative	
Total bilirubin (mg/dl) 0.3–1.2	0.5	0.3
Aspartate aminotransferase (IU/L) < 31	38	57
Alanine aminotransferase (IU/L) > 34	20	159
Lactate dehydrogenase (U/L) 120–246	252	213
Creatine phosphokinase (U/L) 120–180	130	
Ferritin (ng/mL) 10–291	119	
Serum Creatinine (mg/dL) 0.5–1.1	0.6	0.34
Protein (g/dL) 5.7–8.2	7	
Albumin (g/dL) 3.8–5.4	4.3	
Prothrombin time (sec.) 9.4–12.5	12.9	12.8
Kaolin-kephalin time (sec.) 25.4–36.9	27.4	29.1
Fibrynogen (g/l) 2-3.9	3.4	
d-Dimer (ng/ml) 0-500	7247	987

**Table 2:** Immunological tests

Variables	Result
Anti-SARS CoV-2 in IgM class (> 1)	6.2
Anti-SARS CoV-2 in IgG class (> 7.1)	120
SARS-CoV-2 PCR	Positive
Rapid SARS-CoV-2 antigen test	Negative
Cryoglobulins	Negative
Human Immunodeficiency Virus	Negative
Hepatitis B surface antigen	Negative
Anti-hepatitis C virus	Negative
EBV IgM antibodies	Negative
CMV IgM antibodies	Negative
Influenza A – RNA	Negative
Influenza B – RNA	Negative
RSV – RNA	Negative
ANA	Negative
pANCA	Negative
cANCA	Negative
Anti-CCP	Negative
Rf-IgM	Negative
Complement levels C3 (mg/dl) 90–170	109.2
Complement levels C4 (mg/dl) 12–36	28.7
IgA (mg/dl) 40–350	280
IgM (mg/dl) 50–300	137
IgG (mg/dl) 650–1600	1250

## Search strategy

PubMed, Scopus and Web of Science databases were searched for articles containing case reports published between December 1, 2019 and July 31, 2023 using the following keywords: ‘COVID-19’, ‘SARS-CoV-2’, ‘IgA vasculitis’, ‘Henoch-Schönlein purpura’, and ‘palpable purpura’. Only clinical case studies written in English and accessible in full text were included. The quality of clinical cases were evaluated by Equator network’s Clinical Case Reporting Guideline Development-CARE checklist.

## Discussion

The association between COVID-19 infection and development of autoimmune diseases is widely debated. To date, an increased risk of inflammatory myopathy, systemic scleroderma, and systemic lupus erythematosus has been demonstrated [16–19]. Various forms of systemic vasculitis have also been described in the literature, including several cases of IgAV in patients infected with COVID-19 [20]. IgA vasculitis is a rare and poorly understood systematic vasculitis in adults. The data indicate that clinical presentation and outcome of IgAV in adults differ from children.

We present a case of a 58-year-old woman who developed IgAV with skin, joint and kidney involvement after COVID-19 infection, successfully treated with GCs.

The first case of an IgAV patient was published by Allez et al. It was a 24-year-old man with Crohn’s disease treated with TNFα inhibitors, who developed skin lesions, joint complaints, and abdominal pain during COVID-19 infection. Histopathological examination of the skin confirmed IgAV. In the case described, a link between IgA vasculitis and Crohn’s disease or treatment with TNFα inhibitors could not be ruled out [21]. One of the first cases of IgA nephropathy (IgAN) in the course of COVID-19 infection was described by Suso et al. in a 78-year-old man. Symptoms of vasculitis (skin lesions, arthritis, hematuria, proteinuria, renal failure) occurred e few weeks after COVID-19 [22]. The most data on the co-occurrence of IgAV and COVID- 19 are provided by the works of Faroog et al., Messova et al., Wonga et al. Faroog et al. described 13 patients with IgAV and IgAN associated with COVID-19 infection. The mean age was 23.8 years (range 1–78), the majority of patients were male (77%). Nearly half of the patients (46%) were over 18 years of age. In 10 patients (77%), symptoms of COVID-19 infection and/or positive PCR co-occurred with symptoms of IgAV. In the remaining three patients (23%), COVID-19 infection resolved before the onset of IgAN/IgAV symptoms. The most common clinical manifestations were skin lesions (85%) and gastrointestinal symptoms (62%). Less common were joint pain (54%) and arthritis with swelling (31%). Renal involvement was present in about half of the patients. Most patients received GCs and reported recovery or improvement; 2 patients in early childhood died [23]. Messova et al. analyzed a group of patients infected with COVID-19 between December 1, 2019 and December 1, 2021, who met criteria for IgA vasculitis. Among 13 patients (8 adults and 5 children), 12 patients were male. More than half of the patients had symptoms of kidney damage: proteinuria, hematuria, acute renal failure. Four patients had no respiratory symptoms. In their analysis, Messova et al. showed that all elderly patients had symptoms of kidney damage, and 80% of them developed acute renal failure. The results of this analysis are similar to other studies: COVID-19-associated IgAV mainly affects adults and is characterized by a more severe disease course with skin, joint and kidney damage. A positive effect of GCs has been reported in most individuals [24]. An interesting analysis was conducted by Jedlowski et al. They compared the course of IgAV induced by COVID − 19 in children and adults in a group of 10 patients (5 children, 5 adults). The time from the onset of COVID-19 symptoms to the development of IgAV was 2–37 days. There was no significant difference between the two groups in terms of overall organ involvement, except for joint pain and proteinuria, which were more common in adults. Like other researchers, they showed that COVID-19-associated IgAV compared to classical IgAV was significantly more common in adults and male subjects. As in the population of patients with classical IgAV, the course of the disease in adults is more severe, complicated by renal involvement. The prognosis of IgAV patients with COVID infection is fairly good; GCs are effective in most cases [25].

The course of IgAV in the patient reported here was relatively mild. To date, few descriptions of IgAV in women have been reported, with men predominating in each report. Symptoms of vasculitis appeared several days after the symptoms of COVID-19 infection (similar to the work of Jedlowski et al.). The clinical presentation was dominated by skin lesions, arthritis, and renal involvement (according to Faroog et al., the percentages were 85%, 54%, and about 50%, respectively). COVID-19 infection was complicated by pulmonary embolism. Symptoms of the disease quickly resolved with low-dose GCs, as in previously described cases.

## Conclusions

COVID-19 infection can lead to IgAV, especially in men. Palpable purpura may be the first sign of the disease. Renal complications with proteinuria and renal failure development are more common in adults [24, 25]. Early diagnosis and use of GCs leads to rapid remission of the disease. It is recommended that future studies with larger sample sizes be conducted to further explore the relationship between COVID-19 and IgAV.

## Limitations

The study has limitations due to the small number of clinical cases, making it challenging to draw definitive conclusions or establish a causal relationship between COVID-19 and IgAV. The quality of the work is significantly affected by the absence of a skin biopsy, which was not possible due to the lack of patient consent. Publication bias may be suspected, as clinicians are more likely to report clinically significant and challenging cases. As case reports make up the majority of the literature, it is difficult to generalise findings to the wider population.
